# Breast Cancer Detection with Reduced Feature Set

**DOI:** 10.1155/2015/265138

**Published:** 2015-05-19

**Authors:** Ahmet Mert, Niyazi Kılıç, Erdem Bilgili, Aydin Akan

**Affiliations:** ^1^Department of Electrical and Electronics, Piri Reis University, 34940 Istanbul, Turkey; ^2^Department of Electrical and Electronics, Istanbul University, 34320 Istanbul, Turkey

## Abstract

This paper explores feature reduction properties of independent component analysis (ICA) on breast cancer decision support system. Wisconsin diagnostic breast cancer (WDBC) dataset is reduced to one-dimensional feature vector computing an independent component (IC). The original data with 30 features and reduced one feature (IC) are used to evaluate diagnostic accuracy of the classifiers such as *k*-nearest neighbor (*k*-NN), artificial neural network (ANN), radial basis function neural network (RBFNN), and support vector machine (SVM). The comparison of the proposed classification using the IC with original feature set is also tested on different validation (5/10-fold cross-validations) and partitioning (20%–40%) methods. These classifiers are evaluated how to effectively categorize tumors as benign and malignant in terms of specificity, sensitivity, accuracy, *F*-score, Youden's index, discriminant power, and the receiver operating characteristic (ROC) curve with its criterion values including area under curve (AUC) and 95% confidential interval (CI). This represents an improvement in diagnostic decision support system, while reducing computational complexity.

## 1. Introduction

Breast cancer is one of the leading causes of death among all cancers for women [1]. Early detection and correct diagnosis of cancer are essential for the treatment of the disease. However, the traditional approach to cancer diagnosis depends highly on the experience of doctors and their visual inspections. Naturally, human beings can make mistakes due to their limitations. Humans can recognize patterns easily. However, they fail when probabilities have to be assigned to observations [2]. Although several tests are applied, exact diagnosis may be difficult even for an expert. That is why automatic diagnosis of breast cancer is investigated by many researchers. Computer aided diagnostic tools are intended to help physicians in order to improve the accuracy of the diagnosis [3–5].

A study was carried out to demonstrate that the machine learning may improve the accuracy of diagnosis. In Brause's work, the result shows that the most experienced physician can diagnose with 79.97% accuracy while 91.1% correct diagnosis is achieved with the help of machine learning [6].

Tumors are classified as benign and malignant. Benign tumors are not cancerous or life threatening. However these can increase the risk of getting breast cancer. Malignant tumors are cancerous and more alarming than benign tumors. Although significant studies are performed for early detection, about 20% of all women with malignant tumors die from this disease [7].

In order to improve accuracy of breast mass classification as benign and malignant, the performance of back-propagation artificial neural network (ANN) was evaluated [8]. Moreover, the fast learning rates and generalization capabilities of radial basis function neural networks (RBFNN) have showed excellent accuracy in microcalcification detection task [9, 10]. The advantages of RBFNN are simple structure, good performance with approaching nonlinear function, and fast convergence velocity. Thus, it has been widely used in pattern recognition and system modeling [11, 12]. On the other hand, the structure of RBFNN increases when the net's input dimension increases. Moreover, the irrelevant components in the inputs will decrease the generalization performance of RBFNN [13].

Support vector machine (SVM) is an effective statistical learning method for classification [14]. SVM is based on finding optimal hyperplane to separate different classes mapping input data into higher-dimensional feature space. SVM has advantage of fast training technique, even with large number of input data [15, 16]. Therefore it has been used for many recognition problems such as object recognition and face detection [17–19].

Principal component analysis (PCA) is a technique to reduce dimensionality using second order statistical information [20]. Independent component analysis (ICA) is a recently developed method in pattern recognition and signal processing fields [21, 22]. It involves higher order statistics to extract independent components that involve richer information than PCA. ICA can be used to reduce dimensionality before training *k*-NN, ANN, RBFNN, and SVM. Consequently the complexity of classifiers can be reduced; convergence velocity and performance can be increased [13, 23].

The objective of the proposed study is to analyze the effect of feature reduction using ICA on classification of the tumors as benign or malignant. Thus, the dimension of WDBC dataset is reduced into only one feature using ICA. The reduced data is subdivided into test and training data using 5/10-fold cross-validation and 20% partitioning to evaluate the performance of *k-*NN, ANN, RBFNN, and SVM. Performance measures including accuracy, specificity, sensitivity, *F*-score, Youden's index, and discriminant power are computed and the receiver operating characteristic (ROC) curve is plotted to compare the classifiers.  Section 2 summarizes background knowledge on dataset, ICA, *k*-NN, ANN, RBFNN, SVM, and performance measures. In Section 3, the methodology deployed in this study is described. In Sections 4 and 5 experimental results are presented and discussed. Finally, there is a conclusion part in Section 6.

## 2. Materials and Methods

### 2.1. Dataset Information

**Figure 1:**
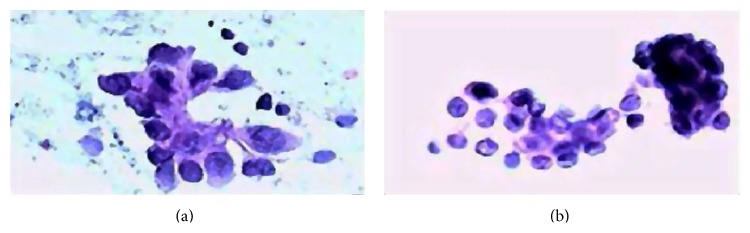
FNA biopsies of breast. Malignant (a) and benign (b) breast tumors [24].

WBDC dataset includes 569 instances with class distribution of 357 benign and 212 malignant. Each sample consists of ID number, diagnosis (B = benign, M = malignant), and 30 features. Features have been computed from a digitized image of a fine needle aspirate (FNA) of a breast mass shown in Figure 1.

Ten real-valued features given in Table 1 calculated for each cell nucleus, and the mean, standard error, and “worst” or largest (mean of the three largest values) of these features were calculated for each image, resulting in 30 features [24].

### 2.2. Independent Component Analysis

The basic model of ICA is as follows. Suppose that the observed signal is the linear combination of two independently distributed sources. The observed signal can be written as follows:

(1) $$\begin{array}{c} x=As, \end{array}$$

where *s* is a vector that consists of the source signals, *A* is an unknown mixing matrix composed of constant elements, and *x* is a vector of observed values. The unknown mixing matrix, *A*, is estimated using the ICA, and then separating matrix *W* is computed which is the inverse of *A*. The original signal can be found by

(2) $$\begin{array}{c} \hat{s}=Wx. \end{array}$$

The computing of the independent components (ICs) begins with centering data by removing the mean values of the variable, as in principal component analysis (PCA). Whitening, also known as sphering data, is the next step. Data which have been whitened are uncorrelated (as PCA). On the other hand, all variables have variances of one. PCA can be used for both these computations because it decorrelates the data and gives information on the variance of the decorrelated data in the form of the eigenvectors [25]. ICs are determined by applying a linear transformation to the uncorrelated data:

(3) $$\begin{array}{c} \text{i}{\text{c}}_{i}=b_{i}^{T}x, \end{array}$$

where ic is the independent component and *b* is the vector to reconstruct ic. There are many different approaches to estimate *b* using an objective function that relates to variable independence. In this study, FASTICA algorithm has been used to compute ICs, due to its flexibility and interactive mode [26].

### 2.3. Artificial Neural Networks

**Figure 2:**
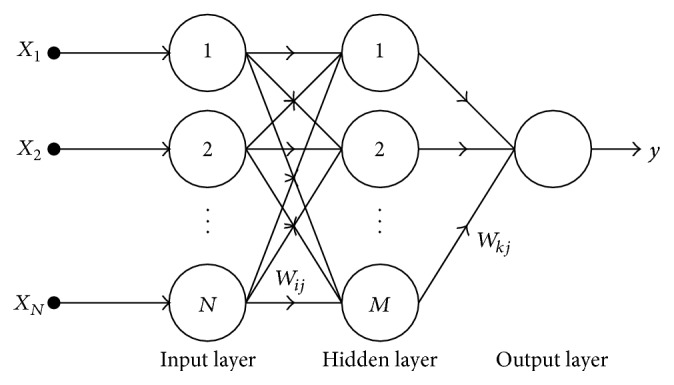
Architecture of feedforward neural network.

Feedforward neural network (FFNN) is most popular ANN structure due to its simplicity in mathematical analysis and good representational capabilities [27, 28]. FFNN has been used successfully to various applications such as control, signal processing, and pattern classification. FFNN architecture is shown Figure 2.

*N* states the number of input patterns and *M* states the number of neurons in hidden layer. Neurons in the hidden layer receive weighted inputs from a previous layer and transfer output to the neurons in the next layer in FFNN, and these computations can be described as

(4) $$\begin{array}{c} y_{\text{net}}=\overset{n}{\underset{i=1}{\sum }}x_{i}w_{i}+w_{0},\\ y_{\text{out}}=f\left(y_{\text{net}}\right)=\frac{1}{1+e^{-y_{\text{net}}}},\\ E=\frac{1}{2}\overset{k}{\underset{i=1}{\sum }}{\left(y_{\text{obs}}-y_{\text{out}}\right)}^{2}, \end{array}$$

where *w*_0_ is bias, *w*_*i*_ is the weight of each input neuron, *x*_*i*_ is input neuron, *y*_net_ is composed of the summation of weighted inputs, *y*_out_ is the output of system, *f*(*y*_net_) denotes the nonlinear activation function, *y*_obs_ is the observed output value of neural network, and *E* is the error between output value and network result [29].

A RBFNN also consists of feedforward architecture with three layers, but the hidden layer uses Gaussian function mostly and is called radial basis layer. Each neuron consists of a radial basis function (RBF) centered on a point. The centers and spreads are computed by the training. A hidden neuron computes the Euclidean distance of input vector and the test case from the neuron's center point. Thus, it applies the RBF kernel function to the distance using the spread values.

### 2.4. Support Vector Machine (SVM)

SVM is a supervised learning algorithm studied for data classification and regression. It was proposed by Boser et al. [30] and Vapnik [31]. SVM algorithm is used to find a hyperplane that separates the classes minimizing training error and maximizing the margin in order to increase generation capability.

**Figure 3:**
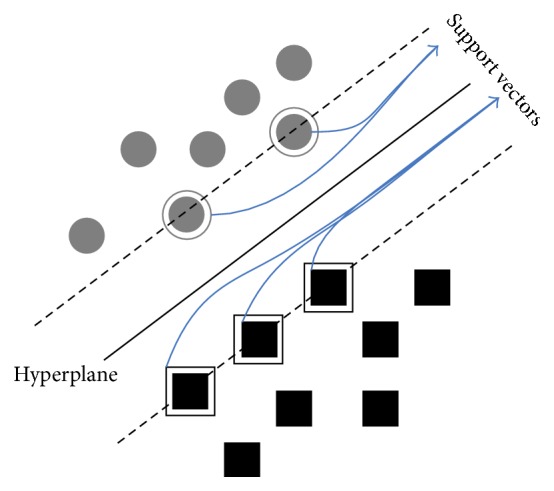
The separating hyperplane with support vectors.

When the datasets are linearly separable, a linear SVM algorithm can be used to classify them. The algorithm tries to maximize the margin. Support vectors are the points lying on the margins that are shown in Figure 3.

The discriminant function of the hyperplane can be described by the following equation:

(5) $$\begin{array}{c} g\left(x\right)=w^{T}x+b, \end{array}$$

where *x* describes data points, *w* is a coefficient vector, and *b* shows offset from the origin. In case of linear SVM *g*(*x*) ≥ 0 for the closest point on the one of the class, *g*(*x*) < 0 for the closest point belongs to another class. Margin (2/‖*w*‖^2^) should be maximized for better generalization ability minimizing the cost function as follows:

(6) $$\begin{array}{c} J\left(w\right)=\frac{1}{2}{\left\|w\right\|}^{2} \end{array}$$

*y*_*i*_(*W* ^*T*^ *x*_*i*_ + *b*) ≥ 1  *i* = 1,2,…, *n* and *y*_*i*_ = {+1, −1} denotes class labels.

This is a quadratic optimization task with respect to a set of linear inequality constraints. From Karush-Kuhn-Tucker (KKT) conditions the Lagrange function is found by

(7) $$\begin{array}{c} L_{p}\left(w,b,\alpha \right)=\frac{1}{2}{\left\|w\right\|}^{2}-\overset{n}{\underset{i=1}{\sum }}\alpha_{i}\left\{y_{i}\left(W^{T}x_{i}+b\right)-1\right\}, \end{array}$$

where *α*_*i*_ are Lagrange multipliers and *L*_*p*_ must be minimized to find out optimal *w* and* b*. The optimization equation can be written as

(8) $$\begin{array}{c} \text{Maximize}\left[\overset{n}{\underset{i=1}{\sum }}\alpha_{i}-\frac{1}{2}\overset{n}{\underset{i,j=1}{\sum }}\alpha_{i}\alpha_{j}y_{i}y_{j}x_{i}^{T}x_{j}\right]. \end{array}$$

The other usage of SVM is that it can solve nonlinear classification problems through the trick of a kernel function. The kernel function maps data points onto a higher-dimensional space in order to construct a hyperplane separating the classes. The new discriminant function is found by

(9) $$\begin{array}{c} g\left(x\right)=W^{T}\Phi \left(X\right)+b, \end{array}$$

where Φ(*X*) represents the mapping of input vectors, onto the kernel space *X*. Therefore, the optimization equation can be written as:

(10) $$\begin{array}{c} \text{Maximize}\left[\overset{n}{\underset{i=1}{\sum }}\alpha_{i}-\frac{1}{2}\overset{n}{\underset{i,j=1}{\sum }}\alpha_{i}\alpha_{j}y_{i}y_{j}K\left(x_{i},x_{j}\right)\right], \end{array}$$

where *K*(*x*_*i*_, *x*_*j*_) is the kernel function equals to {Φ(*x*_*i*_), Φ(*x*_*j*_)}. The kernel functions can be radial basis function (RBF), polynomial or any symmetric functions which satisfy the Mercel conditions [32].

### 2.5. Performance Measures

There are several ways to evaluate the performance of classifiers. Confusion matrix keeps the correct and incorrect classification results to measure the quality of the classifier.  Table 2 shows a confusion matrix for binary classification, where TP, TN, FP, and FN denote true positive, true negative, false positive, and false negative counts, respectively.

The most common empirical measure to assess effectiveness is the accuracy for classifier and it is calculated by

(11) $$\begin{array}{c} \text{Accuracy}=\frac{\text{TP}+\text{TN}}{\text{TP}+\text{TN}+\text{FP}+\text{FN}}. \end{array}$$

Sensitivity measures the proportion of actual positives which are correctly identified and specificity measures the proportion of negatives which are correctly identified. These are formulated by

(12) $$\begin{array}{c} \text{Sensitivity}=\frac{\text{TP}}{\text{TP}+\text{FN}},\\ \text{Specificity}=\frac{\text{TN}}{\text{TN}+\text{FP}}. \end{array}$$

*F*-score is a measure of test accuracy. It considers both precision and the recall to compute. These are calculated by

(13) $$\begin{array}{c} \text{precision}=\frac{\text{TP}}{\text{TP}+\text{FP}},\\ \text{recall}=\frac{\text{TP}}{\text{TP}+\text{FN}},\\ F\text{-Score}=\frac{\left(\beta^{2}+1\right)\times \text{precision}\times \text{recall}}{\beta^{2}\times \text{precision}+\text{recall}}, \end{array}$$

where *β* is the bias and *F*-Score is balanced when *β* = 1. It favors recall when *β* < 1 and favors precision otherwise.

Other two measures which are used to analyze the performance of a classifier in medical diagnosis are discriminant power (DP) and Youden's index. DP evaluates how well a classifier distinguishes between positive and negative samples:

(14) $$\begin{array}{c} \text{DP}=\frac{\sqrt{3}}{\pi }\left(\log X+\log Y\right), \end{array}$$

where

(15) $$\begin{array}{c} X=\frac{\text{sensitivity}}{1-\text{sensitivity}},\;\;Y=\frac{\text{specificity}}{1-\text{specificity}}. \end{array}$$

The result can be summarized as follows: DP < 1 then “poor discriminant,” DP < 2 then “limited discriminant,” DP < 3 then “fair discriminant” and other cases then “good discriminant.” Youden's index evaluates a classifier's ability to avoid failure [33] and is described as

(16) $$\begin{array}{c} \gamma =\text{sensitivity}-\left(1-\text{specificity}\right). \end{array}$$

Youden's index is used summary measure of the receiver operating characteristic (ROC) curve. The diagnostic performance of a test or a classifier to distinguish diseased cases from normal cases is evaluated using the ROC curve analysis [34].

In this study, an attempt has been made to evaluate the performance of the classifiers computing the aforementioned measures for 5/10-fold cross-validations (CV) and 20% data partitioning. For 5-CV or 10-CV, the data are divided into 5 or 10 subsets, and each subset is sequentially deployed as test data while others are deployed as trainig data. Thus 5 or 10 iterative processes are evaluated to determine distinguishing capability of the classification model. Data partitioning is easier and less reliable than CV method. In our simulations, once 20% of the data is randomly selected as test data, the other samples are used for training.

## 3. Methodology

**Figure 4:**
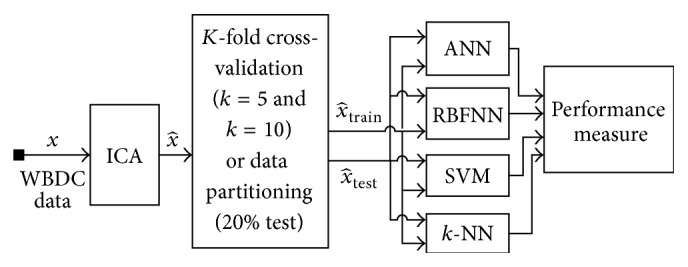
The basic model of the study.

In this study, the original 30 features of WDBC data and reduced one feature using ICA are deployed to evaluate the classifiers performance on breast cancer decision. Thus, the proposed model shown in Figure 4 is applied to WDBC data that have 30 features and 569 instances (patients) were used to train and test the models.

First, the dimensionality of the data is reduced using ICA and partitioned into subsamples using 5/10-CV and 20% partitioning to evaluate the classifiers. The subsamples have been used sequentially to train and test ANN, RBFNN, SVM, and *k-*NN. The outputs of the classifiers have been evaluated to find out performance measures.

**Figure 5:**
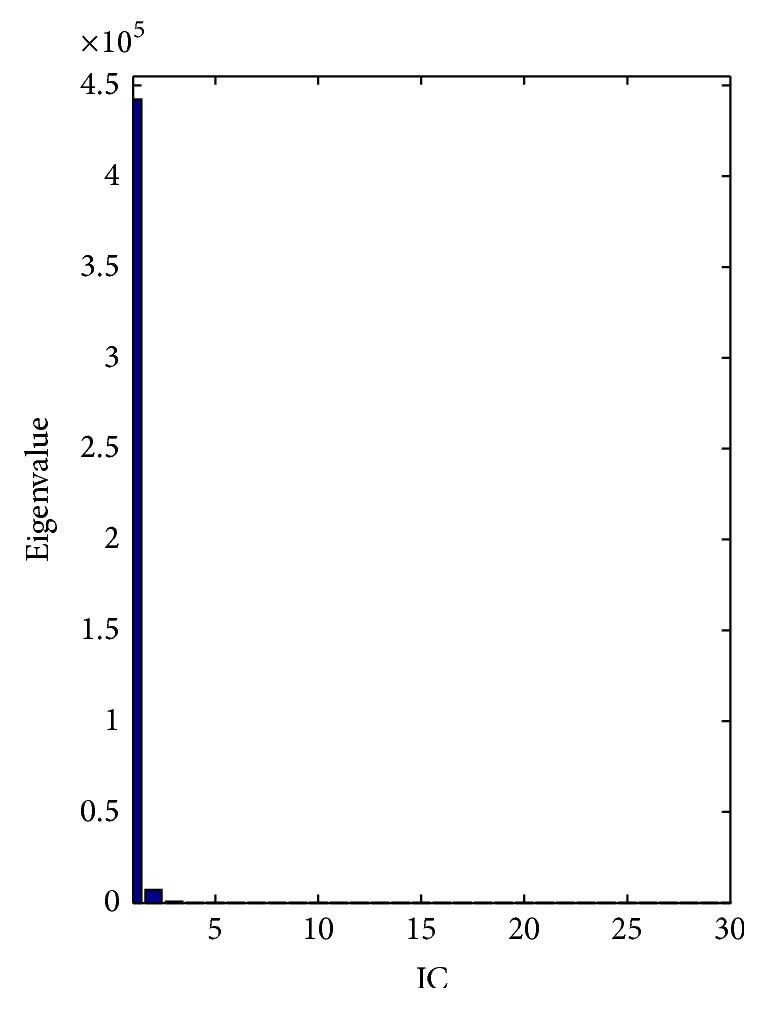
Corresponding eigenvalues of the WDBC data.

First, ICA is used to compute ICs. Since the first IC has distinctly large eigenvalue given in Figure 5, it has been selected as a feature vector.

**Figure 6:**
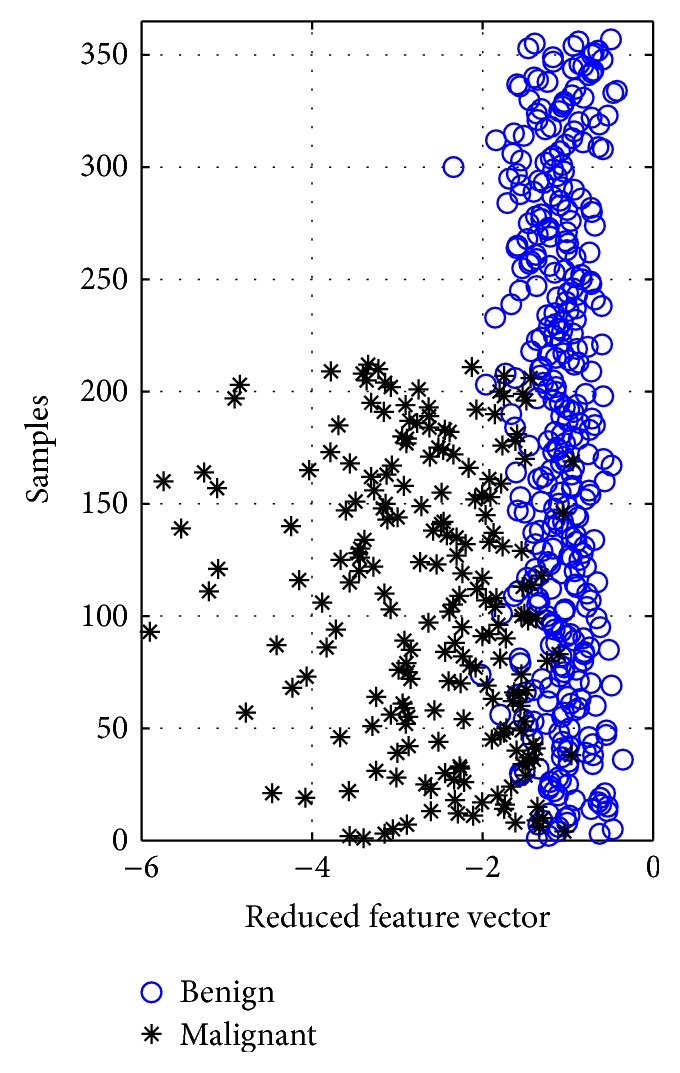
The distribution of computed IC (reduced feature vector).

In other words, one IC can successfully identify the thirty features with the retained 98.205% of nonzero eigenvalues. In addition, the distribution of the IC is given in Figure 6 to indicate its distinguishing capability.

The data are divided into subsets using 5/10-CV and 20% partitioning to test and train classifiers. After training process, the test data are used to evaluate diagnostic performances of the classifiers in terms of sensitivity, specificity, accuracy, *F*-score, Youden's index, DP, and ROC curve.

For training processes, *k*-NN classifier, one-dimensional Euclidean distance, $d=\sqrt{{\left(x_{\text{test}}-x_{\text{training}}\right)}^{2}}$ between test and training samples [35]. The results of *k*-NN classifier are obtained for the *k* values from 1 to 25, and then the performance measures at the best *k* value are stored. The model of ANN is selected as feedforward neural network with one hidden layer. The total number of neurons in the hidden layer is sequentially increased to find the maximum accuracy. Moreover, the activation function of the hidden layer of the network has been chosen as log-sigmoid transfer function. In order to train the network, gradient descent with momentum and adaptive learning rate backpropagation algorithm is used. RBFNN is also evaluated varying the spread value (*σ*). For SVM, linear, quadratic, and RBF kernels are used to explore which type of separating hyperplane is more suitable for breast cancer classification.

## 4. Results

One-dimensional feature vector of WDBC data reduced using ICA is used for training and testing the classifiers. The accuracy, sensitivity, and specificity of one dimensionality have been performed using 5/10 CV technique and 20% of data as test data. Also, the success of the breast cancer classification is generally evaluated on the basis of sensitivity value because the classifying of the malignant mass is more important than the benign mass.

**Figure 7:**
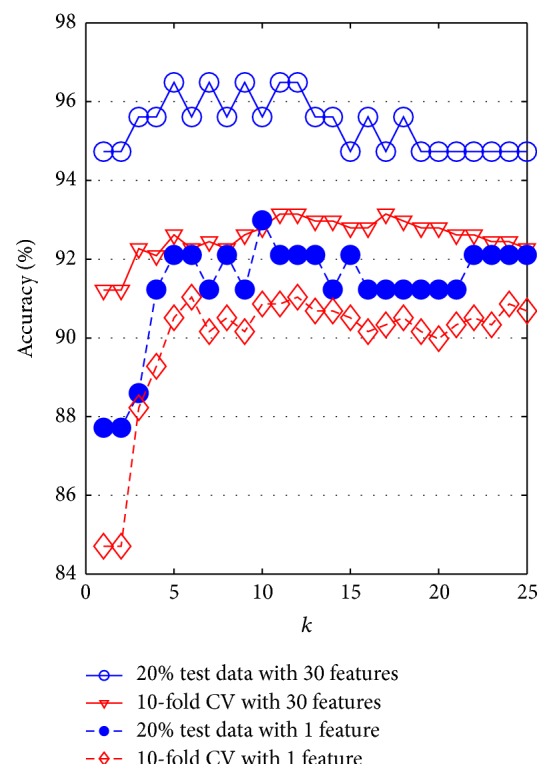
The graph of accuracy of *k*-NN classifier.

The accuracy of the *k-*NN classifier has been computed for varying *k* values between 1 and 25. The comparison graph of the effect of ICA on accuracy of *k*-NN classifier is shown in Figure 7.

The maximum accuracy results when 20% test data with 30 features is 96.49% where *k* = 5. However, reduced one feature vector using ICA provides the accuracy of 92.98% where *k* = 5 and 20% test data is selected. Moreover, the accuracy of *k*-NN classifier is decreased from 93.15% (30 features) to 91.04% (1 feature by ICA) when 10-CV is used to test and train.

**Figure 8:**
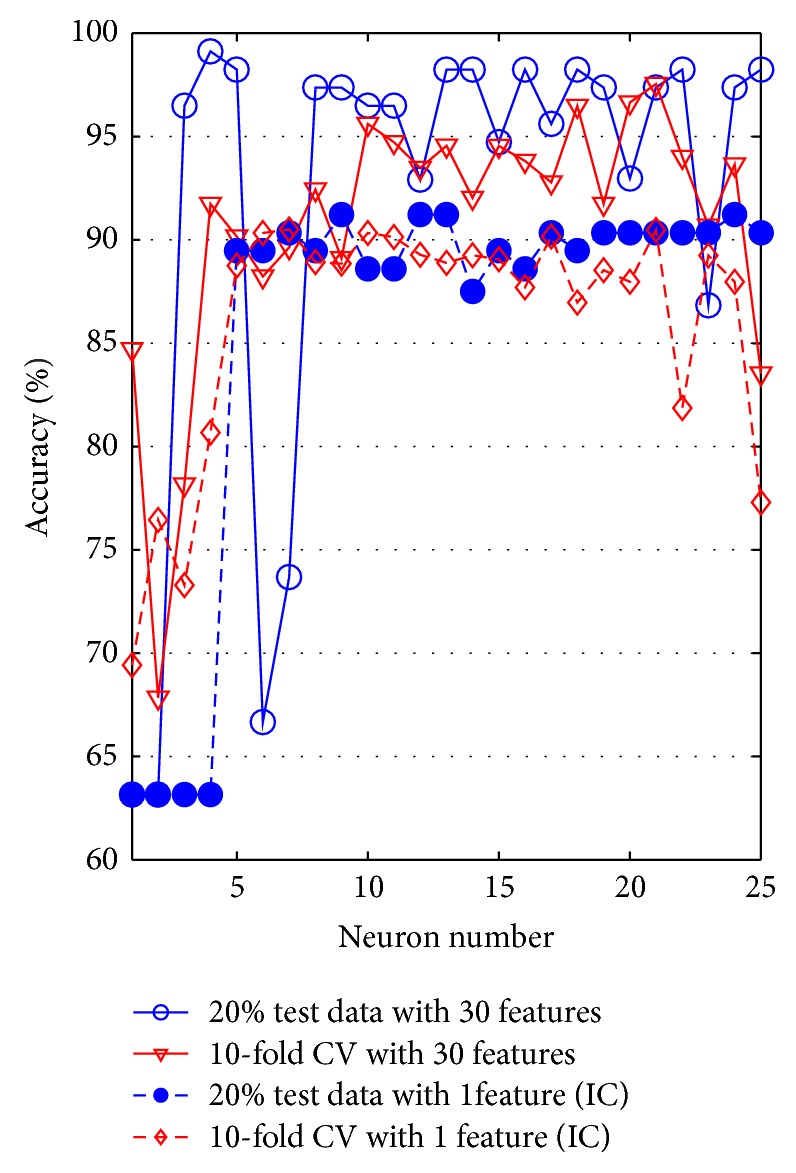
The accuracy graph of ANN.

Accuracy graph of ANN has been plotted varying neuron numbers in the hidden layer for 10/5-CV and 20% test data. The accuracy graph of ANN classifier is given in Figure 8.

ANN classifier has nearly perfect accuracy value of 99.12% (the number of neurons is four) when original 30 features and 20% test data are selected. The effect of ICA on reducing into one feature is changed accuracy value to 91.23% where neuron number is nine. In addition, the accuracy value is changed from 97.54% to 90.51% when 10-CV is used.

**Figure 9:**
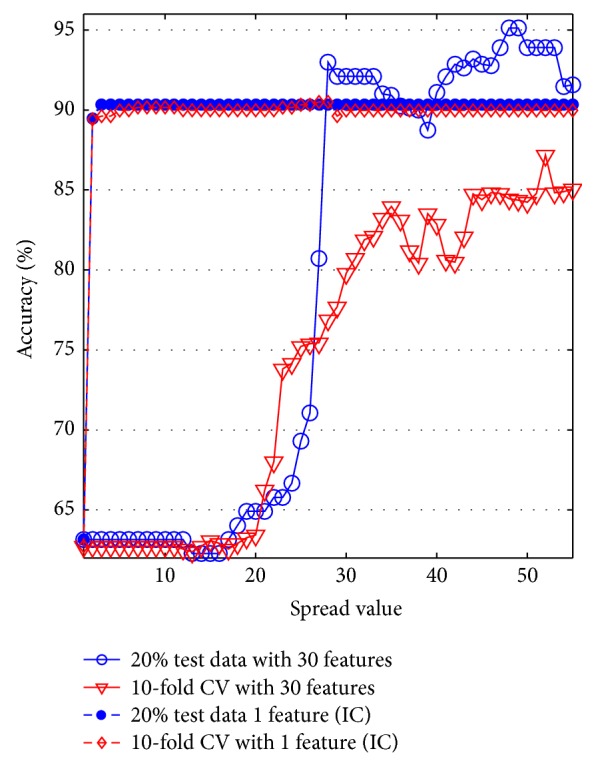
The accuracy graph of RBFNN.

Spread value of RBFNN is adjusted between 0 and 60 to get maximum accuracy for 20% test data ratio and 10/5-CV. The accuracy graph of RBFN is shown in Figure 9.

Referring to the accuracy graph of RBFNN, maximum accuracy, 95.12%, is obtained where spread value is 48 for 20% test data. This value is decreased to 90.35% when reduced one-dimensional feature vector by ICA is used. However, when 10-CV is used, the effect of ICA increases the accuracy from 87.18% (with 30 features) to 90.49% (with 1 feature reduced by ICA).

**Figure 10:**
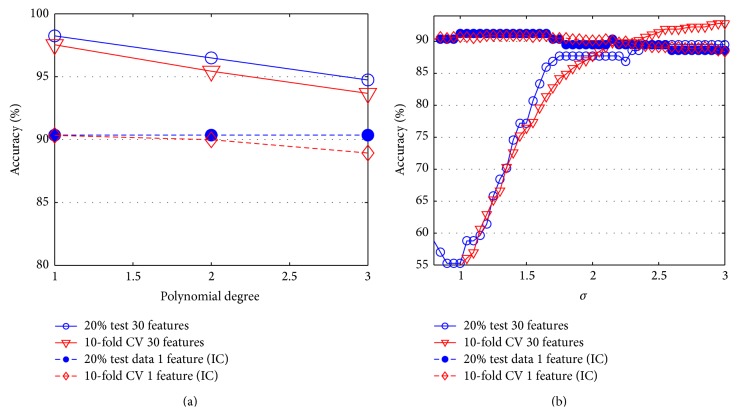
The accuracy graphs of SVM classifiers.

Accuracy evaluation of SVM has been computed for kernel functions including linear, polynomial, and RBF with kernel function parameters such as RBF sigma value for RBF kernel and polynomial degree for polynomial kernel. The accuracy graph of SVM classifier is presented in Figure 10 where the axes of polynomial degree indicate linear kernel when its value equals one.

Generally, SVM classifier with linear kernel provides more accurate result than polynomial and RBF kernel. Its accuracy is 98.25% for 30 features and 90.35% for reduced 1 feature when 20% of data is used as test data. In contrast to polynomial kernel, effect of ICA increases the accuracy of SVM with RBF kernel from 89.47% (30 features) to 91.23% (1 feature). When 10-CV is used, the accuracy is decreased from 97.54% (30 features, linear kernel) and 95.25% (30 features, RBF kernel) to 90.33% and 90.86% (reduced 1 feature by ICA).

*k*-NN, ANN, RBFNN, and SVM have been tested and trained to find out maximum accuracy adjusting their parameter. The performance measures such as accuracy, specificity, sensitivity, *F*-score, Youden's index, and discriminant power of the classifiers are compared to each other. The parameters of the classifiers which provide maximum accuracy are selected to be compared to the other classifiers. In addition to these performance measures, the ROC curve of three classifiers is plotted to enhance visuality of the comparison.

10-CV and one-dimensional feature vector reduced by ICA are used to compare the performances of classifiers. In input data of classifiers, the test data are compared to the original class label to find out TP, TN, FP, and FN values. These values for classifiers are given in the form of confusion matrix in Table 3.

RBFNN classification using 30 original features provides worse performance than reduced one-dimensional feature vector; refer to Table 3. The other classification used with 30 features has slightly higher true values when compared to classification with one feature reduced by ICA.

The performance measures of *k*-NN, ANN, RBFNN, and SVM classifiers such as sensitivity, specificity, accuracy, *F*-score, discriminant power (DP), and Youden's index are given in Table 4 to compare the effect of ICA on the classification.

Discriminant power evaluates how well a classifier distinguishes between positive and negative samples. DP of ANN and SVM with 30 original features differs from 3 which means good discriminant. When ICA is used to reduce to one dimensionality, DP falls to 2.769 (SVM) and 2.655 (ANN). In other words, discriminants turn to fair.

**Figure 11:**
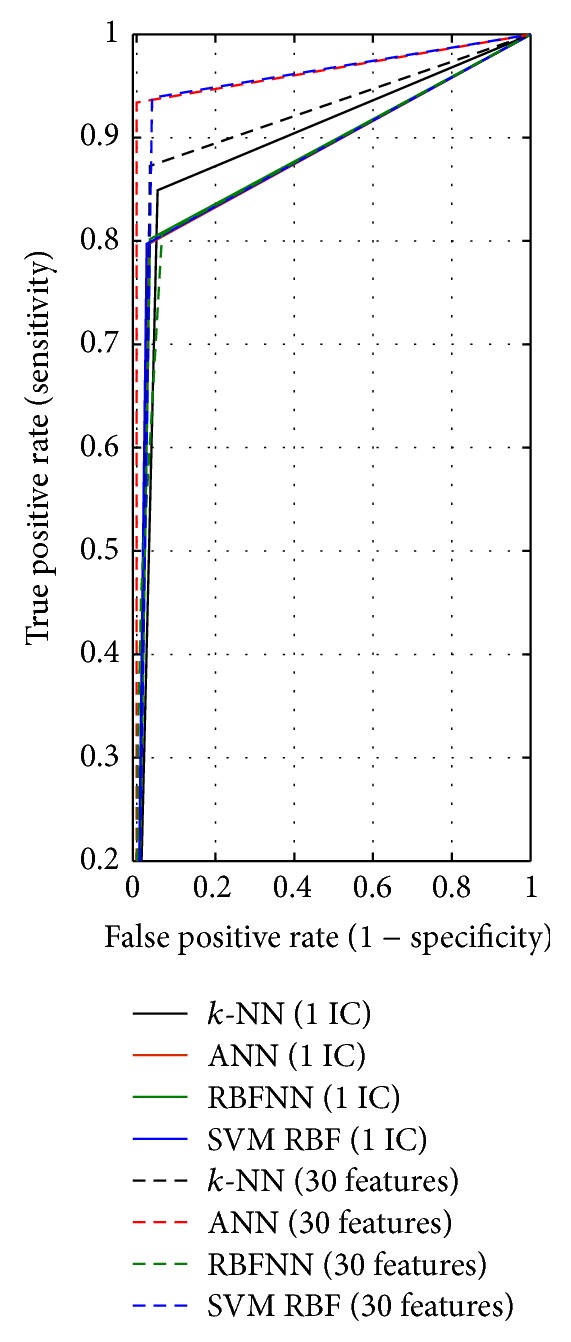
The ROC curves of *k*-NN, ANN, RBFNN, and SVM classifiers.

A higher value of Youden's index shows better ability to avoid failure. *k*-NN results in the highest value of Youden's index; refer to Table 4. Youden's index is used to plot the ROC curve of a classifier. The true positive rate (sensitivity) is plotted in function of the false positive rate (1 − Specificity) for cut-off points in a ROC curve. The ROC curve can be used to compute area under the ROC curve (AUC) and 95% confidence interval (CI). AUC equals 1 when all test data is assigned to true class labels. Higher AUC indicates that higher accuracy 95% CI is another indicator of the ROC curve which can be used to test whether a classifier can distinguish the classes. If its value is not 0.5, it means the classifier can distinguish the classes. The ROC curves of the *k-*NN, ANN, RBFNN, and SVM classifiers using one-dimensional feature vector reduced by ICA and 30 features are presented in Figure 11.

The criterion values of the ROC curves of classifiers are given in Table 5. AUC of the ANN (0.966) and SVM (0.949) results in higher value when 30 original features are used. However, when classification with 1 feature reduced by ICA is evaluated, *k*-NN (0.897) and SVM (0.885) result in higher AUC. It means *k-*NN and SVM classifiers using reduced one feature distinguish samples more correctly.

Table 5 shows that the accuracy of the *k-*NN (91.03%) is better than the accuracy of ANN, RBFNN, and SVM (90.50%, 90.49%, and 90.86%). Generally, one feature reduced by ICA decreases the accuracy of *k*-NN, ANN, and SVM. However, it increases the accuracy of RBFNN.

The aforementioned classification methods are analyzed in terms of computing time given in Table 6 to compare the computational complexities to the classifications with the original 30 features.

The proposed methods have lower computing time when compared to classification of the original dataset. In case of neural network classifications with 30 features, network constructions consume highly more time than classification with one IC. The measured durations of 13.9 and 20.03 seconds are decreased to 11.12 and 14.9 seconds when ANN and RBFN with 20% partitioning are deployed. Particularly, the effect of using IC as feature on complexity is existed when 10-CV is selected. The consumed time of the ANN and RBFNN is decreased from 118.21 and 129.84 seconds to 76.72 and 90.49 seconds, respectively. In addition, ICA decreases computational times of the SVM and *k*-NN classifications, but the rates are less than the neural networks.

## 5. Discussions

**Figure 12:**
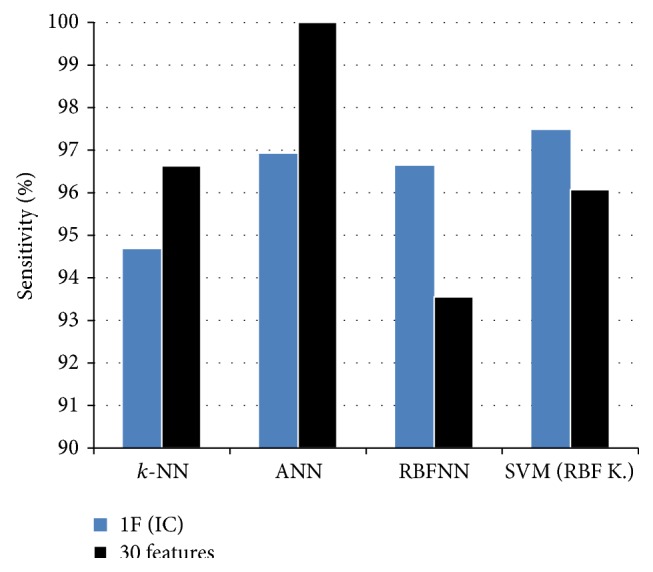
Sensitivity measures of the classifiers.

Sensitivity/specificity indicates the proportion of actual positives/negatives which are correctly identified. While use of one-dimensional feature vector reduced by ICA decreases accuracy slightly, it increases sensitivity values of SVM and RBFNN classifiers. The maximum sensitivity measure belongs to SVM with RBF kernel when one feature is used. The graph of the effect of ICA on sensitivity measures of classifiers is shown in Figure 12.

Sensitivity refers successfully identified malignant samples in cancer classification. Thus, higher sensitivity means higher diagnostic capability of malignant tumors and it can be used to help physicians to diagnose cancerous mass more correctly. The accuracy and sensitivity measures of previous classification studies and this study on WDBC dataset are given in Table 7 to compare the effect of feature reduction using ICA. It should be noted that the studies on WDBC differ from studies on WBC dataset which consists of 699 instances with 10 attributes.

Higher number of features used to classify breast cancer as benign and malignant resultsin slightly higher accuracy. Feature reduction into one using ICA decreases the accuracy of *k-*NN, ANN, and SVM slightly. However, it increases the accuracy of RBFNN and the sensitivity values of SVM and RBFNN.

Referring to Table 7, the sensitivity measures of the classifiers used with one-dimensional feature vector reduced by ICA in this study perform better than the other studies. However, accuracy rates of the proposed classifications (90.53% ± 0.34) are lower than the previous methods (94.93% ± 2.07). The study of WDBC data creators [39] set has the highest accuracy (97.50%) using multisurface method tree (MSM-T) with 3 selected features. Similarly, hybrid methods are more successful than the others. Breast cancer classifications using probabilistic neural network (PNN) with hybrid feature reduction using discrete wavelet transform (DWT) and ICA [40] or classification using SVM with 6-dimensional feature space obtained by *K*-means algorithm [41] have accuracy rates of 96.31% and 97.38% for 10-CV. Particularly, SVM based studies [36, 38] with 30 features have near scores to our one-dimensional results.

## 6. Conclusions

In this study, the effect of dimensionality reduction using independent component analysis (ICA) on breast cancer decision support systems with several classifiers such as artificial neural network (ANN), *k*-nearest neighbor (*k*-NN), radial basis function neural network (RBFNN), and support vector machine (SVM) is investigated. The results of the applied original thirty features of Wisconsin diagnostic breast cancer (WDBC) are compared with the reduced one dimension by ICA. The accuracy rates of the classifications with thirty original features except RBFNN have slightly decreased from 97.53%, 91.03%, and 95.25% to 90.5%, 91.03%, and 90.86%, respectively. However, the one-dimensional feature vector causes RBFNN classifier to be more distinguishing with the increased accuracy from 87.17% to 90.49%. Furthermore, the sensitivity rates which define the successfully recognized malignant samples are increased from 93.5% to 96.63% for RBFNN and from 96.07% to 97.47% for SVM, while the others have slight decrease at the rate between 0.96% and 3.09%. If the objective is to increase the rate of the successfully identified malignant breast cancer using RBFNN or decrease computational complexity without loss of the high accuracy rate, feature reduction applying ICA can be a high performance solution.

## Figures and Tables

**Table 1:** Real-valued features computed for each cell nucleus.

Number	Ten real-valued features
1	Radius (mean of distances from center to points on the perimeter)
2	texture (standard deviation of grey-scale values)
3	Perimeter
4	Area
5	Smoothness (local variation in radius lengths)
6	Compactness (perimeter^2^/area − 1.0)
7	Concavity (severity of concave portions of the contour)
8	Concave points (number of concave portions of the contour)
9	Symmetry
10	Fractal dimension (“coastline approximation” − 1)

**Table 2:** A confusion matrix for binary classification.

Actual value	Recognized value	
	Positive	Negative
Positive	TP	FN
Negative	FP	TN

**Table 3:** The confusion matrices of the classifiers using reduced one dimensionality by ICA (1F denotes one feature and 30F denotes original features).

*k*-NN classifier (*k* = 6)					ANN classifier (neuron number 7)				
	Recognized value					Recognized value			
Actual value	Malignant		Benign		Actual value	Malignant		Benign	
	1F	30F	1F	30F		1F	30F	1F	30F
Malignant	338 (TP)	346	19 (FN)	11	Malignant	346 (TP)	357	11 (FN)	0
Benign	32 (FP)	28	180 (TN)	184	Benign	43 (FP)	14	169 (TN)	198

RBFNN classifier (spread = 28)					SVM classifier (*σ* = 1.3)				
	Recognized value					Recognized value			
Actual value	Malignant		Benign		Actual value	Malignant		Benign	
	1F	30F	1F	30F		1F	30F	1F	30F

Malignant	345 (TP)	334	12 (FN)	23	Malignant	348 (TP)	343	14 (FN)	9
Benign	43 (FP)	138	169 (TN)	74	Benign	43 (FP)	13	169 (TN)	199

**Table 4:** The comparison of ICA algorithm's effect on the classifiers' performance measures (sensitivity, specificity, accuracy, and *F*-score in %).

Measures	*k*-NN		ANN		RBFNN		SVM (RBF K.)	
	1F	30F	1F	30F	1F	30F	1F	30F
*F*-score	92.98	*94.65 *	92.76	*98.07 *	92.61	*80.57 *	93.04	*96.21 *
DP	2.539	*2.912 *	2.655	*InF *	2.606	*1.131 *	2.769	*3.267 *
*ϒ*	0.795	*0.839 *	0.766	*0.934 *	0.763	*0.284 *	0.772	*0.899 *
Accuracy	91.03	*93.14 *	90.5	*97.53 *	90.49	*87.17 *	90.86	*95.25 *
Specificity	84.9	*87.26 *	79.71	*93.39 *	79.71	*34.9 *	79.71	*93.86 *
Sensitivity	94.67	*96.63 *	96.91	*100 *	96.63	*93.55 *	97.47	*96.07 *

**Table 5:** Criterion values of the ROC curves of *k*-NN, ANN, RBFNN, and SVM.

Criterion	*k*-NN		ANN		RBFNN		SVM	
	1F	30F	1F	30F	1F	30F	1F	30F
AUC	0.880	0.911	0.879	0.956	0.881	0.877	0.879	0.945
95% CI	0.86–0.92	0.89–0.94	0.85–0.91	0.94–0.98	0.85–0.91	0.85–0.91	0.85–0.91	0.92–0.97

**Table 6:** CPU time for classification.

Classifier	Partitioning	IC (seconds)	30 features (seconds)
*k*-NN	20%	8.02	8.31
	10-CV	13.52	14.77

ANN	20%	11.12	13.9
	10-CV	76.72	118.21

RBFNN	20%	14.9	20.03
	10-CV	90.49	129.84

SVM (poly)	20%	7.17	7.28
	10-CV	7.47	9.13

SVM (RBF)	20%	9.02	43.30
	10-CV	10.72	19.05

**Table 7:** Comparison of the methods and accuracy of previous studies and this study.

Author	Method	Feature number	Accuracy	Sensitivity
Krishnan et al. [36]	40% test data, SVM (poly.)	30	92.62%	92.69%
	40% test data, SVM (RBF)		93.72%	94.50%

Bagui et al. [37]	64% test data, *k*-RNN	30	96.00%	95.09%
	64% test data, *k*-RNN	Best 3	98.10%	98.05%

Sweilam et al. [38]	PSO + SVM	30	93.52%	91.52%
	QPSO + SVM		93.06%	90.00%

Mangasarian et al. [39]	10-CV, MSM-T	Best 3	97.50%	—

Mert et al. [40]	10-CV, PNN	3 (2IC + DWT)	96.31%	98.88%
	LOO, PNN		97.01%	97.78%

Zheng et al. [41]	*K*-SVM	6	97.38%	—

This study	10-CV, *k*-NN	1 feature reduced by ICA	91.03%	94.67%
	40% test, *k*-NN		92.56%	94.02%
	10-CV, ANN		90.50%	96.91%
	40% test, ANN		90.89%	97.00%
	10-CV, RBFNN		90.49%	96.63%
	40% test, RBFNN		89.98%	96.01%
	10-CV, SVM (linear)		90.33%	96.35%
	40% test, SVM (linear)		90.01%	95.00%
	10-CV, SVM (quadratic)		89.98%	95.24%
	40% test, SVM (quadratic)		91.01%	96.42%
	10-CV, SVM (RBF)		90.86%	97.47%
	40% test, SVM (RBF)		91.03%	97.56%
